# Characterization of Phenolic Acid Antimicrobial and Antioxidant Structure–Property Relationships

**DOI:** 10.3390/pharmaceutics12050419

**Published:** 2020-05-02

**Authors:** Jingyi Liu, Changling Du, Henry T. Beaman, Mary Beth B. Monroe

**Affiliations:** Department of Biomedical and Chemical Engineering, Syracuse University, Syracuse, NY 13244-1200, USA; jliu135@syr.edu (J.L.); cdu108@syr.edu (C.D.); htbeaman@syr.edu (H.T.B.)

**Keywords:** phenolic acids, antimicrobial, antioxidant

## Abstract

Plant-derived phenolic acids (PAs) are small molecules with antimicrobial, antioxidant, anti-inflammatory, and pro-coagulant properties. Their chemistry enables facile potential incorporation into biomaterial scaffolds to provide naturally-derived functionalities that could improve healing outcomes. While PAs have been previously characterized, their structure-property relationships in terms of antioxidant and antimicrobial properties are not well-understood, particularly in the context of their use in medical applications. To that end, a library of PAs with varied pendant groups was characterized here. It was found that increasing the number of radical-scavenging hydroxyl and methoxy groups on PAs increased antioxidant properties. All PAs showed some antimicrobial activity against the selected bacteria strains (*Escherichia coli*, *Staphylococcus epidermidis* (native and drug-resistant), and *Staphylococcus aureus* (native and drug-resistant)) at concentrations that are feasible for incorporation into polymeric biomaterials. In general, a trend of slightly decreased antimicrobial efficacy with increased number of pendant hydroxyl and methoxy groups was observed. The carboxylic acid group of a selection of PAs was modified with a polyurethane monomer analog. Modification did not greatly affect antioxidant or antimicrobial properties in comparison to unmodified controls, indicating that the carboxylic acid group of PAs can be altered without losing functionality. These results could be utilized for rational selection of phenolic moieties for use as therapeutics on their own or as part of a biomaterial scaffold with desired healing outcomes.

## 1. Introduction

A majority of hospital-acquired infections are related to medical devices. In a 1999 study, 95% of cases of urinary tract infections were catheter-related, and 87% of bloodstream infections originated from a vascular catheter [1]. In addition to difficulties in treating infections, bacterial colonization can negatively affect the function of implanted devices. For example, vascular occlusion is associated with infection of central venous catheters, and bacteria on joint prostheses and dental implants can cause loosening and failure [2,3,4]. An added complication of infection is the grand challenge of antibiotic-resistant bacteria strains, which is caused in part by antibiotic overuse. Antibiotic resistant infections contribute to ~100,000 deaths each year in the US, and “superbugs” are expected to cause more deaths than all cancers combined by 2050 if no effective alternative treatments are developed [5,6]. Based on these trends, implantable medical devices that are resistant to infection could provide enhanced safety and efficacy, and non-drug-based antimicrobials must be pursued to reduce these risks and complications.

Honey has been used for centuries in wound care, and recent clinical data indicates that honey-based wound dressings promote healing and reduce infection [7,8]. These positive outcomes are partly attributed to the presence of phenolic acids (PAs) [9]. PAs exhibit broad antimicrobial properties and have been shown to be effective against multi-drug resistant organisms (MDROs) [9,10,11]. PAs are small, simple molecules that contain a carboxylic acid group on their non-active end. This group enables their potential incorporation into a range of polymeric medical devices, including polyurethanes [12]. PAs are secondary metabolites of plants that kill microorganisms and/or inhibit the growth of bacteria as part of the plant antimicrobial mechanism. Inhibitory mechanisms of PAs on bacteria growth are wide-ranging and include destabilizing the bacteria cytoplasmic membrane, altering the permeability of the bacteria plasma membrane, inhibiting extracellular microbial enzymes, directly altering microbial metabolism, and depriving microbes of substrates required for growth [13]. Specifically, PAs can change bacterial physicochemical surface properties. For example, ferulic acid has been shown to decrease hydrophobicity of *Pseudomonas aeruginosa* [14]. PA treatment can also alter bacterial polarity by changing bacteria surface electron acceptors on both gram-positive (increased acceptor components) and gram-negative (decreased acceptor components) strains [14]. It has been shown that as PA concentrations increase, the percentage of cell membrane damage significantly increases, as indicated by release of intracellular K^+^, with greater effects observed with gram-negative bacteria strains than with gram-positive bacteria strains [14].

In addition to their antimicrobial properties, PAs contain hydrogen-donating groups that can react with oxidants to form resonance-stabilized phenoxyl radicals and provide antioxidant properties that could further improve healing outcomes [10,15,16,17]. Although reactive oxygen species (ROS) can be beneficial for healing, prolonged exposure of acute and chronic wounds to high levels of ROS causes cell damage and inhibits wound closure [18]. An excessive amount of ROS is present in chronic wounds, and antioxidants have been shown to be effective at removing ROS and interrupting the chronic inflammatory cycle [18]. In addition, ROS contribute to a highly oxidizing environment for traumatic wounds, and drastic increases in ROS production after injury can cause an oxidative stress response that leads to critical illness (e.g., organ dysfunction, disseminated intravascular coagulation, and acute phase response) [17,19,20,21]. Honey-based PAs provide a potential free radical scavenger that could block ROS-induced cytotoxicity by decreasing lipid peroxidation and DNA damage [18].

PAs show promise as multifunctional, bioinspired molecules on their own or as biomaterial additives to reduce device-associated and drug-resistant infections and improve healing outcomes. A number of studies have been performed on PA antimicrobial and antioxidant characterization (Table 1); however, many studies focus on the use of PAs in relation to food-borne illnesses and plant pathogens rather than common bacteria in hospital-acquired infections and wound bacteria, such as *Escherichia coli* (*E. coli*), *Staphylococcus epidermidis* (*Staph. epi.*), and *Staphylococcus aureus* (*Staph. aureus*). Many PAs have not been characterized with drug-resistant strains, and some of the literature data is conflicting (e.g., ferulic and vanillic acids were determined to be effective against *E. coli* in studies performed by Merkl et al. but not in those performed by Chatterjee et al.) [10,22]. Finally, a connection between PA structure and function (i.e., antioxidant activity and antimicrobial efficacy) is minimal. Campos et al. showed that hydroxycinnamic acids induce greater ion leakages and higher proton influx than hydroxybenzoic acids, but this study was carried out with wine lactic acid bacteria, which may not be relevant for therapeutic use of PAs [23]. While a significant amount of data on PA properties is available in the literature, Table 1, their potential therapeutic use is currently hindered by the disparate information and the large number of PAs available for use. A systematic study on PA antimicrobial and antioxidant properties in addition to cytocompatibility measurements could provide a valuable framework for future generations of biomaterials with PA-based functionality.

**Figure 1:**
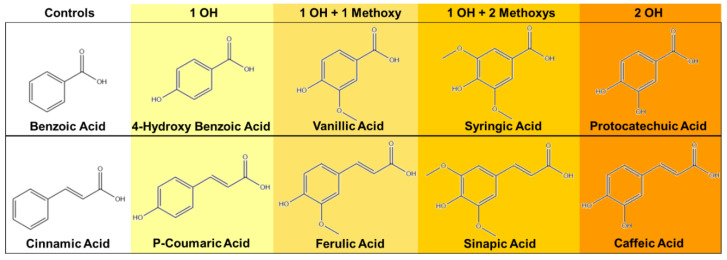
Chemical structures of 10 selected phenolic acids (PAs). Top row: benzoic acid (BA)-based PAs; bottom row: cinnamic acid (CA)-based PAs.

The goal of this work is to characterize a library of PAs and establish their structure–property relationships. Ten PAs were selected to characterize in terms of antimicrobial and antioxidant properties, Figure 1. The PAs without pendant hydroxyl (OH) groups on the ring structures (benzoic acid (BA) and cinnamic acid (CA)) are not expected to demonstrate antioxidant properties. Additional BA- and CA-based PAs were chosen with varied pendant groups (1 OH group, 1 OH group with 1 or 2 methoxy groups, and 2 OH groups). Antioxidant properties are hypothesized to be tied to pendant group chemistry, and all selected PAs are expected to have varying degrees of antimicrobial efficacy. To enhance clinical relevancy of data available on PAs, their antimicrobial activity was characterized against *E. coli*, native and drug-resistant *Staph. epi.*, and native and drug-resistant *Staph. aureus*. This study fills in gaps in understanding of the properties of the PAs with pendent group variations, providing relationships between structure and properties by screening the 10 selected PAs in terms of antioxidant properties and antimicrobial efficacy against five common pathogenic wound and hospital-acquired infection bacteria. An additional consideration is reaction of the carboxylic acid group on PAs during incorporation into polymeric biomaterials and its potential effects on PA functionality. To address this issue, selected PAs were modified with polyurethane monomer analogs and compared to their unmodified counterparts. This research will aid in the rational design of PA-based treatment strategies and PA-containing biomaterials with desired functionality.

## 2. Materials and Methods

### 2.1. Materials

All chemicals were purchased from Fisher Scientific (Waltham, MA, USA) unless otherwise specified. Cell and bacteria strains were purchased from ATCC (Manassas, VA, USA).

### 2.2. Modified Phenolic Acid (MPA) Synthesis

**Figure 2:**
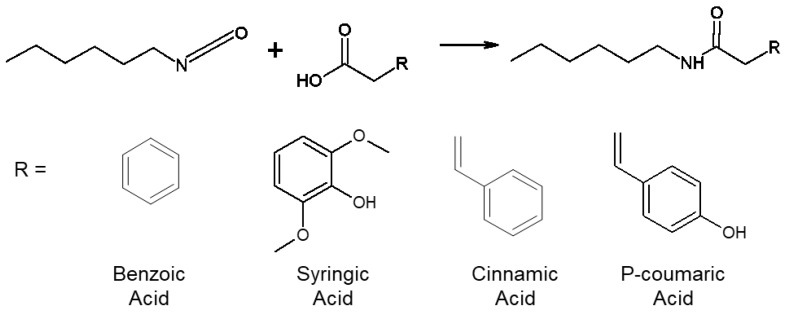
Synthesis of modified phenolic acids to serve as analogs for phenolic acids after incorporation into a polyurethane biomaterial.

MPAs were synthesized according to the scheme shown in Figure 2. Approximately 3 g of each PA was dried under vacuum overnight and dissolved in anhydrous dimethyl sulfoxide (DMSO) in a reaction flask at ~0.1 g/mL. Hexyl isocyanate (HI) (1.1 molar equivalents) was added dropwise to the reaction under anhydrous conditions. The solution was reacted under nitrogen at 65 °C for ~72 h or until the isocyanates were fully reacted, as indicated by a disappearance of the isocyanate peak at ~2250 cm^−1^ in the Fourier transform infrared spectroscopy (FTIR) spectra of the reaction contents.

Complete reaction resulted in separation of the reaction contents into a “viscous” and “liquid” portion after cooling to room temperature. Gravity filtration was utilized to separate the viscous and liquid portions. The product was precipitated from the filtered liquid portion in cold water at a 1:5 volume ratio (liquid portion:cold water), filtered, and dried under vacuum overnight. The MPA structure was confirmed using FTIR spectroscopy (amide formation: shift of C=O peak from ~1670 cm^−1^ to ~1610 cm^−1^, introduction of NH peak at ~3310 cm^−1^; HI addition: introduction of methyl peaks at ~2870–2950 cm^−1^).

### 2.3. Antioxidant Characterization: Hydrogen Peroxide Scavenging

The antioxidant capacity of the PAs and corresponding MPAs was measured based on their H_2_O_2_ scavenging capabilities as previously described [30]. All selected PAs and MPAs were dissolved in DMSO at 5 mg/mL. A 0.002% H_2_O_2_ solution was prepared in water, a 0.1M phosphate-buffered saline (PBS) solution was prepared in water, dying agents (phenol red dye and horseradish peroxidase) were dissolved in PBS at 0.2 mg/mL and 0.1 mg/mL, respectively, and a 1M sodium hydroxide solution was prepared in water. Each PA and MPA sample was serially diluted from 5 mg/mL to 0.078 mg/mL in 10 μL DMSO in triplicate in a 96-well plate. Blank solutions (10 uL DMSO) were also included in each plate. Then, 10 μL of the prepared H_2_O_2_ solution and 80 μL of PBS were added to each sample well. After 10 min of incubation at 37 °C, 100 μL of the prepared dying agents was added. Subsequently, the plate was incubated for 15 min in at 37 °C, and 5 μL of the sodium hydroxide solution was added to each sample well. Hydrogen peroxide scavenging was analyzed immediately after addition of sodium hydroxide using a plate reader (FLx800, Bio-Tek Instruments, Inc.) at an absorbance of 610 nm (optical density (O.D.) 610). The antioxidant properties were quantified in terms of H_2_O_2_ scavenging activity (*H_s_*) using Equation (1),

(1) $$H_{s}=100\%\times [C_{0}-(C_{PA}\times C_{0})],$$

where *C*_0_ is the absorbance value of the control group with no PA and *C_PA_* is the absorbance value of the PA sample at the specific concentration.

### 2.4. Antioxidant Characterization: Cell Protection from Hydrogen Peroxide

#### 2.4.1. Cell Culture

NIH/3T3 Swiss mouse fibroblasts (ATCC–CCL92) were used for in vitro cell culture. Cells were cultured at 37 °C/5% CO_2_ with Dulbecco’s Modified Eagle Medium (DMEM, high glucose GlutaMAX, Life Technologies, Inc., Carlsbad, CA, USA) supplemented with 10% heat-inactivated fetal bovine serum (FBS, Life Technologies, Inc.) and 1% penicillin-streptomycin (Gibco). Cells were used between passages 4 and 6 after three days of culture. For all cytocompatibility studies, cells were seeded into the wells of a 96 well tissue-culture polystyrene plate at 10,000 cells/well and cultured for 24 h. Cell morphology was observed microscopically using a Zeiss Axiovert inverted microscope to confirm even cell distribution before testing. Media was removed, and cells were washed with sterile phosphate buffered saline (PBS) prior to testing.

#### 2.4.2. PA Cytocompatibility

PAs were dissolved in sterile DMSO at 100 mg/mL and then diluted to 1 mg/mL in cell culture media. PA solutions were added to each cell-containing well in triplicate. Controls included media-only, media with DMSO (positive/cytocompatible control), and 70% ethanol (negative/cytotoxic control). After 3 h and 24 h of exposure, a neutral red uptake (NRU) assay was run to quantify cytocompatibility. Solutions were removed from wells and replaced with a neutral red solution. After 3 h of incubation with neutral red, the cells were fixed with 0.1% CaCl_2_ in 0.5% formaldehyde. The neutral red dye was then solubilized in 1% acetic acid in 50% ethanol for measurement. A plate reader (FLx800, BioTek Instruments, Inc., Winooski, VT, USA) was used to measure absorbance at 540 nm (*Abs*_540_). Cell viability was calculated according to Equation (2),

(2) $$Cell\;Viability\left(x\right)=\frac{Abs_{540}\left(x\right)}{Abs_{540}\left(control\right)}\times \;100\%,$$

where *x* is the selected treatment group and the DMSO control is used as a standard that equals 100% viability.

#### 2.4.3. Hydrogen Peroxide Toxicity

H_2_O_2_ was serially diluted from 30% to 0% in PBS and then diluted to 5% to 0% in cell culture media. H_2_O_2_ solutions were added to each cell-containing well in triplicate. Controls included media-only, media with PBS (positive/cytocompatible control), and 70% ethanol (negative/cytotoxic control). After 3 h of exposure, an NRU assay was run, and cell viability for each concentration of hydrogen peroxide was calculated as described above. Viability versus H_2_O_2_ concentration was plotted, and a best fit line was utilized to determine the concentration of H_2_O_2_ that killed ~60–70% of cells, which was measured as 40.5 μM.

#### 2.4.4. PA Protective Effects

PA solutions were prepared as described in Section 2.4.2 in a solution of 40.5 μM H_2_O_2_ in media and added to cell-containing wells in triplicate. Positive controls included media-only and media with DMSO, and the negative control was 0.75% H_2_O_2_ in media. After 3 h of exposure, an NRU assay was run to quantify cell viability as described above using the DMSO control as the 100% viability standard.

### 2.5. Antimicrobial Characterization

#### 2.5.1. Preparation of Bacteria Strains

*Escherichia coli* (*E. coli,* 397E strain), *Staphylococcus epidermidis* (*Staph. epi*., native (FDA strain PCI 1200) and drug-resistant (4483 strain: multi-drug resistant)) and *Staphylococcus aureus* (*Staph. aureus*, native (Wichita strain) and drug-resistant (F-182 strain: methicillin and oxacillin resistant)) were utilized to test the efficacy of PA antimicrobial properties. Before incubating with PAs, bacteria strains were grown in 5 mL of fresh LB broth (prepared at 25 g/L of deionized water and autoclaved) at 37 °C for ~16–17 h. Subsequently, 1 mL of the 5 mL bacteria medium was removed and cultured in 9 mL of fresh LB broth until bacteria reached the logarithmic growth period when optical density at an absorbance of 600 nm (O.D. 600) equals 0.6. The O.D. value was measured using a plate reader (FLx800, Bio-Tek Instruments, Inc.).

#### 2.5.2. DMSO Bacterial Toxicity

Before testing PA antimicrobial performance, each bacteria strain was cultured with a range of concentrations of DMSO to ensure that DMSO does not affect their growth and interfere with measurements. Different volumes (0, 1, 2, 4, 8, 16, 32, 64, and 128 μL) of DMSO were added to LB broth (total volume of 200 µl/well) in a 96 well plate in triplicate. Then, 20 μL of the prepared log-phase growth bacteria medium was added into each well. The plate was incubated with shaking at 37 °C for 24 h. Bacteria growth was analyzed via plate reader absorbance readings at O.D. 600 at 0, 1, 2, 4, and 24 h with a goal of finding the maximum volume of DMSO that could be used in PA testing without affecting the growth of bacteria (comparable O.D. 600 value to 0 μL samples). From these tests, it was determined that up to 5 μL of DMSO could be utilized with *E. coli* without affecting growth and up to 10 μL of DMSO could be utilized with *Staph. epi.* and *Staph. aureus* strains (native and drug-resistant) without affecting growth.

#### 2.5.3. PA IC50 and Log Reduction

PAs were serially diluted in triplicate from 5 to 0.078 mg/mL in 5 μL DMSO and 95 μL of fresh LB broth for *E. coli* tests and in 10 μL DMSO and 90 μL of fresh LB broth for *Staph. epi.* and *Staph. aureus* tests. Controls included wells with 5 μL or 10 μL DMSO in 95 μL or 90 μL of fresh LB broth (DMSO control), 100 μL LB broth (LB control), and a 1% penicillin-streptomycin solution in 5 μL or 10 μL DMSO and LB broth (drug control). For the drug-resistant strains, 1% methicillin or oxacillin solution was prepared in DMSO and LB broth as additional drug controls. Twenty microliters of prepared log-phase growth bacteria medium and 100 μL fresh LB broth were added to all wells in the plate. Bacterial growth was analyzed via absorbance readings at O.D. 600 over 24 h and quantified in terms of IC50 and log reduction values.

*IC*_50_ is the measure of the concentration of antimicrobial agent required to kill 50% of bacteria. It illustrates potency of inhibiting bacteria growth and provides information on minimal concentrations required for future incorporation into biomaterial scaffolds. At each time point, PA concentration was plotted against O.D. 600 values, and a best-fit line was applied to the linear curves. *IC*_50_ was calculated using Equations (3) and (4):

(3) $$\mathrm{Half}\;\mathrm{concentration}=\frac{C_{0}-C_{1}}{2},$$

(4) $$IC_{50}=\frac{\mathrm{Half}\;\mathrm{concentration}-b}{a},$$

where *C*_0_ is the O.D. value of DMSO group, *C*_1_ is the O.D. value of the drug control group, b is the y-intercept of the PA concentration curve, and a is the slope of the PA concentration curve.

Log reduction is the measure of how a specified concentration of antimicrobial reduces bacteria concentration and was calculated using Equation (5) for 5 mg/mL samples at each time point.

(5) $$\mathrm{Log}\;\mathrm{reduction}=Log_{10}\left(\frac{C_{0}}{C_{1}}\right),$$

where *C*_0_ is the O.D. value of the DMSO control and *C*_1_ is the O.D. value of a PA.

#### 2.5.4. MPA Colony Forming Unit Quantification

Due to low solubility of MPAs in aqueous solvents, sediment was observed in 5 mg/mL wells, which affected absorbance readings in the multi-well plate assay. To address this issue, colony forming units (CFUs) were quantified with MPAs in comparison to their PA controls. LB-agar (LB at 20 g/L, agar at 15 g/L) was prepared in deionized water, autoclaved, poured into Petri dishes, and allowed to gel overnight. Each selected PA and MPA was dissolved at 100 mg/mL in 20 μL DMSO and mixed with 380 μL fresh LB broth in a 24-well plate. Fresh LB broth (400 μL) and 20 μL DMSO in 380 μL LB broth were used as controls. Subsequently, 40 μL of prepared log-phase growth bacteria medium was added to each well in the plate. Each sample was tested in triplicate.

At 4 h and 24 h, 10 μL of the bacteria medium was extracted from each sample well and diluted by 10,000 in fresh LB. Then, 100 μL of the diluted solution was applied to the surface of LB-agar plates and cultured for 24 h at 37 °C. Photographs were obtained of each plate surface after culturing, and ImageJ software was used to quantify colony forming units (CFUs) and analyze bacteria growth in terms of log reduction using Equation (4), where C_0_ is the CFU of the DMSO control and C_1_ is the CFU of the sample.

### 2.6. Statistical Analysis

ANOVA was used to determine statistical significance in cell and bacteria studies. Tukey’s post hoc test was applied for independent variables with greater than two levels. When comparing two specific samples, statistical analysis was performed by an unpaired two-tailed Student’s *t*-test. Statistical significance was accepted at *p* < 0.05.

## 3. Results and Discussion

### 3.1. PA Antioxidant Properties

In this work, hydrogen peroxide (H_2_O_2_) was utilized as an oxidizing agent to quantify the antioxidant properties of the 10 selected PAs in terms of H_2_O_2_ scavenging activity [15]. In general, reaction of hydroxyl groups with oxidizing agents (e.g., reactive oxygen species) forms resonance-stable phenoxy radicals, which imparts phenols with antioxidant properties [16]. Therefore, we hypothesized that PAs that do not contain pendant hydroxyl (OH) groups (cinnamic acid (CA) and benzoic acid (BA) controls) would not demonstrate antioxidant capability.

**Figure 3:**
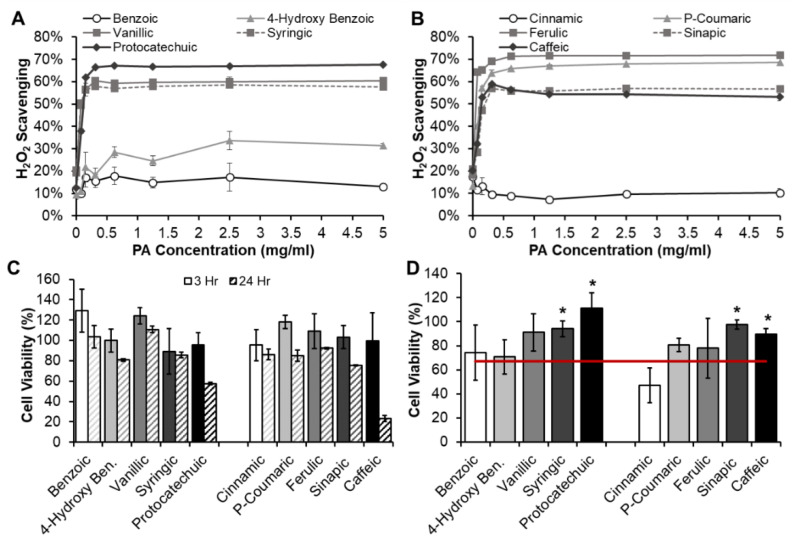
Antioxidant properties of PAs as quantified by hydrogen peroxide (H_2_O_2_) scavenging capabilities from the (**A**) benzoic acid group and (**B**) cinnamic acid group. PA (**C**) 3T3 cytocompatibility and (**D**) protective effects against H_2_O_2_ for 3T3s. Red line: DMSO + H_2_O_2_ control. * Significant increase (*p* < 0.05) in viability relative to DMSO + H_2_O_2_ control. *n* = 3, mean ± standard deviation displayed.

Figure 3 shows the H_2_O_2_ scavenging capabilities of BA- and CA-based PAs. For the BA group, Figure 3A, BA does not demonstrate antioxidant capacity, and 4-hydroxy benzoic acid with only one OH group has relatively low antioxidant capacity, with moderate improvements relative to the BA control. These results are in line with the hypothesis. With the addition of methoxy and additional hydroxyl groups in protocatechuic, syringic, and vanillic acids, antioxidant efficacy is significantly increased. As for the CA group, in Figure 3B, CA does not demonstrate antioxidant capabilities, as expected. The addition of hydrogen donating groups onto the other CA-based PAs resulted in significantly improved antioxidant capabilities. In comparing the BA- and CA-based PAs, it should be noted that the addition of one OH group to CA (P-coumaric acid) resulted in a much higher increase in H_2_O_2_ scavenging than the corollary BA-based 4-hydroxy benzoic acid with one OH group. Beyond this difference, for both BA- and CA-based PAs, the minimum (10–21% and 13–30%, respectively) and maximum (57–68% and 53–72%, respectively) H_2_O_2_ scavenging rates were comparable, indicating that the side chain does not have a major impact on antioxidant properties of PAs. Previous work by Natella et al. compared a library of BAs and CAs using a competition kinetic test with crocin in comparison to Trolox [16]. This work found that in general, CA derivatives had higher antioxidant efficacy than their BA equivalents, and that P-coumaric acid had modest antioxidant activity, particularly in comparison with our data. This discrepancy is likely due to the introduction of crocin as a competitive antioxidant in the previous studies. Our controlled analysis of individual PA effects on H_2_O_2_ scavenging provides an expanded view of the structure/antioxidant properties that can be combined with previous work to rationally choose a PA with desired antioxidant activity.

### 3.2. PA Cytocompatibility and Protective Antioxidant Effect

As an initial indication of PA safety, cytocompatibility of the PA library was measured using 3T3 mouse fibroblasts in a NRU assay (Figure 3C). The FDA defines adequate cytocompatibility of medical devices to be ≥75% [31]. While these studies were conducted on small molecules rather than 3D materials or devices, the ISO standard is the most relevant for consideration of potential biomaterial monomer components. Based upon this standard, all PAs are cytocompatible over 24 h except for protocatechuic and caffeic acids, which both have two pendant hydroxyl groups. While the hydroxyl groups act as radical scavengers, previous work has demonstrated that protocatechuic acid induces oxidative stress at high concentrations (>10 mM) [32]. PAs were tested at ~5.5–8.2 mM here. This result should be considered when designing PA-based therapeutics to ensure that PAs are utilized as concentrations that are active without inducing cytotoxicity. Thus, the full library of PAs with the exception of protocatechuic and caffeic acids (which may have acceptable cytocompatibility at lower concentrations) has potential for use in biomedical applications.

Based on these positive results, the effects of PA antioxidant properties on cells was characterized, Figure 3D. In this study, cells were exposed to a concentration of H_2_O_2_ that killed 60–70% of cells and treated with each PA at 1 mg/mL. Cell viability was measured to determine whether PAs could scavenge H_2_O_2_ in the presence of cells to improve their survival. Similar trends were observed here as in the H_2_O_2_ scavenging study. Namely, BA and CA treatment did not improve cell viability. The addition of only one hydroxyl group with 4-hydroxy benzoic and P-coumaric acids and the addition of one hydroxyl and one methoxy group with vanillic and ferulic acids did not have a significant effect on viability. However, the addition of a second methoxy and/or hydroxyl group with syringic, protocatechuic, sinapic, and caffeic acids provided enough antioxidant activity to significantly increase (*p* < 0.05) cell survival to >90%. Interestingly, protocatechuic acid treatment resulted in the highest viability in this study, despite its reduced cell viability at 24 h. Future studies will focus on determining the “sweet spot” of concentrations of antioxidant PAs with and without oxidative stressors in the presence of cells over varying time frames to better understand this phenomenon. As was observed in Figure 3A, BA and 4-hydroxybenzoic acid had similar antioxidant effects, with an increase (not significant) in activity observed in vanillic acid. Similarly, all CA-based PAs with hydroxyl and/or methoxy functional groups had increased protective effects (*p* < 0.1) relative to CA. No clear trends were observed between BA- or CA-based side chains and antioxidant efficacy in the presence of cells, indicating that the ring structure has a more significant effect on antioxidant activity. Overall, these results provide an initial indication of the potential for PAs with a high number of functional groups to reduce harmfully high ROS levels in the presence of cells to improve healing outcomes.

### 3.3. PA Antimicrobial Properties

#### 3.3.1. *E. coli* (Gram-Negative Bacteria)

**Figure 4:**
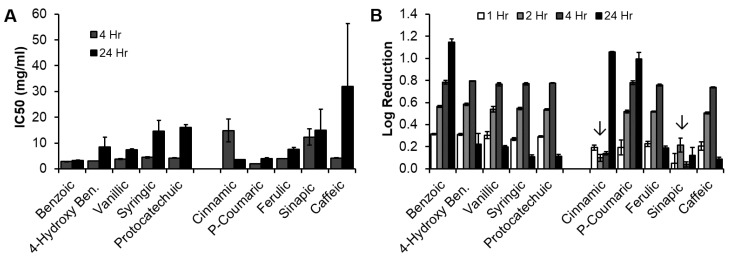
(**A**) IC50 and (**B**) log reduction of PAs against *E. coli. n* = 3, mean ± standard deviation displayed. Downward arrow indicates low solubility of cinnamic and sinapic acids at 5 mg/mL. Cinnamic acid solubility improved by 24 h, while sinapic acid had low solubility over the full 24-h study.

IC50s of the PA library against *E. coli* was calculated at 4 h and 24 h, Figure 4A. With the exception of CA, there was an increase in IC50 between 4 h and 24 h for all PAs (significant increase (*p* < 0.05) for all but 4-hydroxy benzoic, sinapic, and caffeic acids), indicating a reduced antimicrobial efficacy relative to the drug control over time. These reductions in efficacy over time are likely due to bacterial uptake of PAs to reduce effective concentration during live bacterial growth [33]. The significant reduction in IC50 with CA is attributed to solubility. Namely, CA was not fully solubilized in the bacterial media at the 4 h time point, which resulted in sediment in the wells that affected absorbance readings. By 24 h, the CA had fully solubilized, resulting in reduced absorbance and improved readings. It should be noted that this change in solubility over time was only observed in the presence of *E. coli* (i.e., no increase in solubility was observed over 24 h in media without bacteria), indicating that the bacteria alters CA solubility in some way, such as uptake into the cell or enzymatic alterations to the molecule. No consistent trends were observed between the BA and CA groups when comparing PAs with similar ring structures, indicating that the side chain has a negligible effect on efficacy against *E. coli*. However, there was a general trend of decreased efficacy with increased ring side groups at 24 h within both the BA and CA groups. It should be noted that wells that contained caffeic acid experienced a yellow color change in the media at 24 h, which may have affected absorbance readings and could account for its particularly low performance and relatively large error. Previously synthesized polyurethane foams with CA contained between ~5 mg and 20 mg CA/mL (cm^3^) of foam. Thus, all PAs had IC50 values that were in the range for possible incorporation into a biomaterial scaffold at 4 h and/or 24 h.

*E. coli* log reduction was characterized at 1, 2, 4, and 24 h (Figure 4B). When comparing each PA’s maximum log reduction (at 24 h for benzoic, cinnamic, and P-coumaric acids; at 4 h for all others), no trends were observed between PAs in the BA and CA groups that have similar ring side groups. In the BA group, BA was significantly more effective at reducing bacteria growth (*p* < 0.05), and all other PAs induced statistically similar log reductions. CA was the most effective at reducing *E. coli* growth in the CA group. A general trend of decreased efficacy with increased side group was observed when comparing cinnamic, p-coumaric, ferulic, and caffeic acids. Sinapic acid was found to be relatively ineffective in terms of log reduction. This result was replicated in our experiments, but does not correlate well with literature reports of sinapic acid efficacy against *E. coli* [34]. In our experiments, this result is attributed to low solubility of sinapic acid at 5 mg/mL, resulting in sediment that affected absorbance readings. Beyond solubility effects, when considering the wall structure of gram-negative bacteria, the two-layered structure with an outer lipid layer may explain why sinapic acid was found to be relatively ineffective here. Namely, the steric hindrance from the relatively large size of sinapic acid combined with its increased hydrophilicity from OH and methoxy groups may reduce effective interactions with *E. coli*.

#### 3.3.2. *Staph. epi.* (Native and Drug-Resistant, Gram-Positive Bacteria)

**Figure 5:**
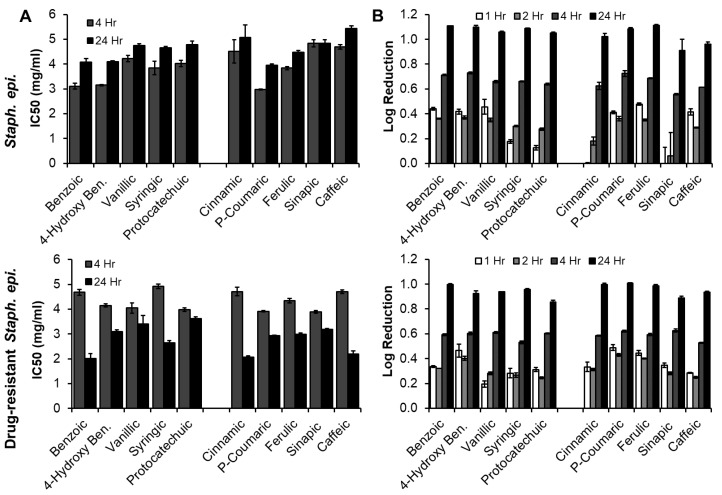
(**A**) IC50 and (**B**) log reduction of PAs against *Staph. epi.* (top row) and drug-resistant *Staph. epi* (bottom row)*. n* = 3, mean ± standard deviation displayed.

The IC50 values of PAs with native and drug-resistant *Staph. epi.* are shown in Figure 5A. With native *Staph. epi.*, a significant increase in IC50 was observed with all PAs except for cinnamic and sinapic acids, which also increased, indicating a reduced efficacy over time relative to the drug control. The opposite trend was observed with drug-resistant *Staph. epi.*, where all PA IC50s decreased between 4 h and 24 h (significant decreases for all but vanillic acid). IC50 values were overall higher for drug resistant *Staph. epi.* at 4 h and lower at 24 h. These results indicates that the drug-resistant strain may be slower to respond to PAs and/or that the drug control (methicillin) has shorter efficacy time frames. There were no trends between BA- and CA-based PAs with similar ring structures, again indicating that the side chain does not affect antimicrobial activity. With native *Staph. epi.*, benzoic and 4-hydroxy benzoic acids had similar IC50s that were lower than vanillic, syringic, and protocatechuic acids with more pendant groups on the rings. CA was the least effective. CA was not visibly insoluble in the studies with *Staph. epi.* that included a higher amount of DMSO. However, it is possible that it still had reduced solubility in these studies, which may have affected its antimicrobial activity. With this exception, there was a general trend of decreased efficacy against *Staph. epi.* with increased ring side groups in the CA group. For the drug-resistant *Staph. epi*. at 24 h, there was again a general trend of decreased efficacy with increased ring side groups in both the BA and CA groups, with the exception of syringic and caffeic acids, which had comparable IC50 values to BA and CA, respectively.

Maximum log reductions were observed at 24 h for all PAs with both *Staph. epi.* strains, Figure 5B. Log reductions were generally lower at all time points for drug-resistant *Staph. epi*. Again, no trends were observed between BA and CA-based PAs with similar ring structures. In the BA group, benzoic and 4-hydroxy benzoic acids had similarly high log reductions of native *Staph. epi.* in comparison with vanillic, syringic, and protocatechuic acids with increased ring side groups. Similarly, cinnamic, p-coumaric, and ferulic acids had similar log reductions that were higher than that of sinapic and caffeic acids. In the drug-resistant strain, BA had the highest log reduction and protocatechuic acid had the lowest log reduction in the BA group. In the CA group, the same trends were observed to those of the native strain: cinnamic, p-coumaric, and ferulic acids had higher log reductions than sinapic and caffeic acids.

#### 3.3.3. *Staph. aureus* (Native and Drug-Resistant, Gram-Positive Bacteria)

**Figure 6:**
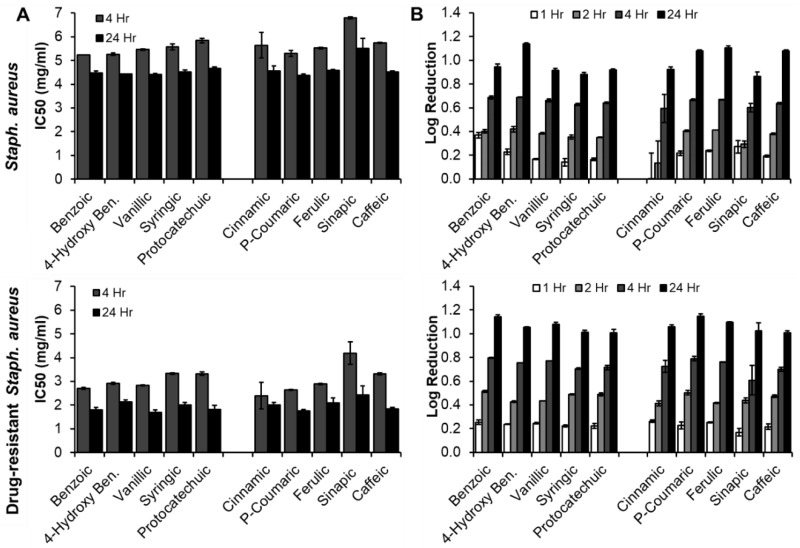
(**A**) IC50 and (**B**) log reduction of PAs against *Staph. aureus* (top row) and drug-resistant *Staph. aureus* (bottom row)*. n* = 3, mean ± standard deviation displayed.

IC50 values of PAs for native and drug-resistant *Staph. aureus* are shown in Figure 6A. All IC50 values dropped between 4 h and 24 h for both bacteria strains, indicating increased efficacy relative to the drug control (penicillin) over time. There were no trends between BA and CA-based PAs with similar ring structures, further confirming that the side chain does not have a significant effect on antimicrobial activity. With the BA group and native *Staph. aureus*, there was a general trend of decreased efficacy with increased BA ring side groups at 4 h, and no trends were observed at 24 h. No clear trends were observed with the CA group at either time point. Overall, the IC50 values for drug-resistant *Staph. aureus* were lower, indicating increased efficacy of PAs against this strain. Again, no clear trends were observed in either group at either time point in relation to ring structures, indicating that the ring pendants do not have a significant effect on efficacy against *Staph. aureus*.

Log reductions are shown in Figure 6B, with all maximum log reductions observed at 24 h for both strains. Similarly to the IC50 measurements, no trends were seen between PAs with similar ring structures from the BA and CA groups. There were no clear trends between ring structures and log reduction for native *Staph. aureus*, but syringic and sinapic acids, with one hydroxyl and two methoxy groups both had the lowest bacterial reduction at 24 h. This result could be attributed to increased steric hindrance with increased bulky groups on the rings that prevents effective interaction with the bacteria. For drug-resistant *Staph. aureus*, a general trend of decreased log reductions with increased ring pendant groups can be seen for both BA and CA-based PAs. When comparing the native and drug-resistant strains, drug-resistant *Staph. aureus* had generally higher log reductions at 4 h and similar log reductions at 24 h.

Three important results that can be gleaned from this data include (1) all PAs show some efficacy against all tested bacteria strains, indicating their potential for use in infection prevention; (2) PA IC50 values are within the range of possible incorporation into biomaterial scaffolds in terms of concentration, enabling potential future synthesis of PA-based antimicrobial materials; and (3) PAs are effective against both native and drug-resistant strains of *Staph. epi.* and *Staph. aureus*. Most of the data indicates that antimicrobial efficacy generally decreases with increased numbers of ring pendant groups. In general, IC50 values were higher and log reductions were lower with *E. coli* in comparison with *Staph. epi.* and *Staph. aureus*, which corresponds with previous work showing that PAs are more effective against gram-positive strains [35]. The uptake of PAs into gram-positive bacteria (*Staph. epi.* and *Staph. aureus*) may be more effective than that into gram-negative *E. coli* due to differences in the cell walls. Namely, gram-negative bacteria have an outer lipid membrane, which may make gram-negative bacteria less likely to interact with relatively hydrophilic agents. This hypothesis correlates with the results of increased efficacy against *E. coli* with reduced number of ring groups, as the hydroxyl and methoxy groups increase hydrophilicity of PAs, thereby reducing potential interactions with the cell wall. Gram-positive bacteria have electron acceptors in their outer membranes that have been previously shown to increase in activity after exposure to PAs [14,36]. As the number of hydroxyl and methoxy side groups increases on PAs, they become less electrophilic, which likely reduces interactions with bacteria and antimicrobial efficacy. While the observed differences in antimicrobial activity with PA structure were generally not significant, the data in this study can be utilized by researchers to rationally select phenolic moieties with desired properties for use an antimicrobial agents.

### 3.4. Modified Phenolic Acid Characterization

#### 3.4.1. Modified Phenolic Acid Antioxidant Properties

**Figure 7:**
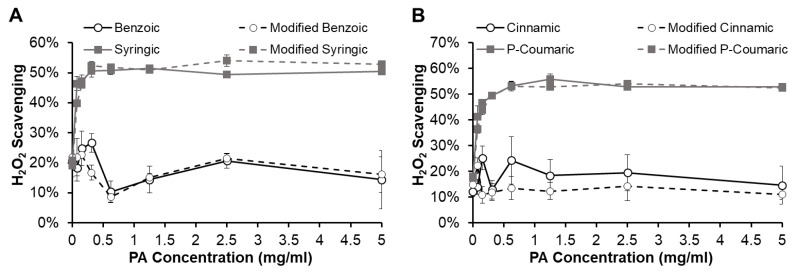
Antioxidant properties of modified phenolic acids in comparison to their unmodified controls as quantified by hydrogen peroxide scavenging capabilities from the (**A**) benzoic acid group and (**B**) cinnamic acid group. *n* = 3, mean ± standard deviation displayed.

The antioxidant properties of modified PAs (MPAs) were characterized to test whether PAs maintain their antioxidant capacity after the chemical modification required for incorporation into polyurethanes (i.e., reaction of the carboxylic acid with an isocyanate to form an amide). Figure 7 shows the H_2_O_2_ scavenging capabilities of representative MPAs, modified benzoic and syringic acids from the BA group (Figure 7A) and modified cinnamic and P-coumaric acids from the CA group (Figure 7B). Modified BA retains low antioxidant capacity at levels that are similar to those of BA. Similarly, syringic acid and modified syringic acid have comparably high antioxidant efficacy. The CA group shows the same trend in that CA and modified CA both have low H_2_O_2_ scavenging capabilities, while P-coumaric acid and modified P-coumaric acid have similarly increased antioxidant properties. These results indicate that the chemical modification of PAs that is required for their incorporation into polyurethanes will not alter their antioxidant capacity, enabling potential incorporation of PAs into antioxidant biomaterial scaffolds. Previous work by Merkl et al. found similar results with esterification of a small selection of this PA library (vanillic, protocatechuic, ferulic, and caffeic acids), wherein antioxidant properties were relatively stable following addition of alkyl groups of varying lengths [10].

Excess ROS can prevent effective wound healing; antioxidant biomaterials could help overcome this problem. In addition to promoting enhanced healing, antioxidant monomers could provide polymeric medical devices with extended biostability in various potential applications. For example, additives in polyurethanes that have ROS scavenging capabilities have been shown to inhibit oxidative degradation, which causes scission and crosslinking of polyurethane chains [37]. The addition of antioxidant PAs into a polymer network provides a new tool to control biomaterial degradation. Beyond wound healing and degradation control, antioxidant PAs have further potential healing benefits. Studies have shown that the onset of a number of diseases, including rheumatoid arthritis, atherosclerosis, and cancer, is related to the presence of free radicals and excess ROS. In future studies, PA-containing scaffolds could be used to reduce the concentration of these compounds as part of a treatment regime [38]. Alternatively, a low level of ROS can be beneficial in healing to reduce infection, regulate healing and angiogenesis, and break-down foreign material [39]. Thus, non-antioxidant PAs could be selected for use in applications where maintenance of ROS levels is desired.

#### 3.4.2. Modified Phenolic Acid Antimicrobial Properties

**Figure 8:**
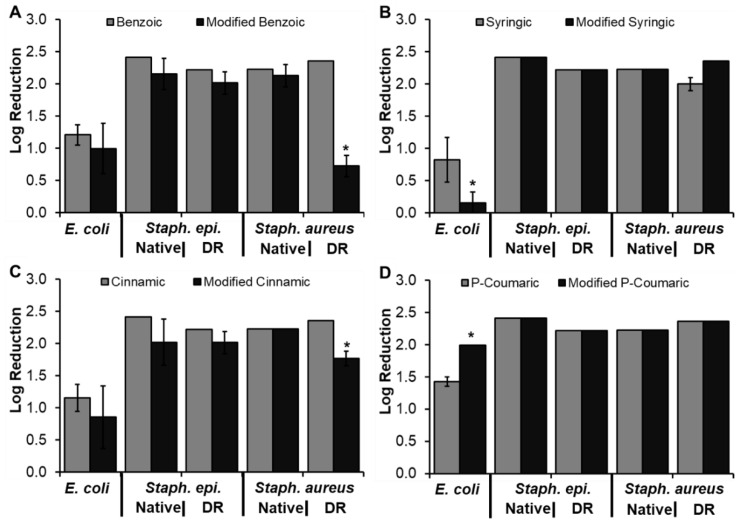
Antimicrobial properties of modified (**A**) benzoic, (**B**) syringic, (**C**) cinnamic, and (**D**) P-coumaric acids in comparison to their unmodified controls. Antimicrobial properties were quantified by log reduction in colony forming unit counts. * *p* < 0.05 relative to unmodified control. *n* = 3, mean ± standard deviation displayed.

Previously, CA was incorporated into polyurethane foams, providing a material with tunable and clinically-relevant thermal and shape memory properties and antimicrobial efficacy against *E. coli* and *Staph. epi*. [12]. These results provide a proof-of-concept that PAs do not need to be released or solubilized from a polymer network to be functional and indicates that reaction of the carboxylic acid does not affect antimicrobial function. To expand upon this work, antimicrobial efficacy of a selection of MPAs (modified benzoic, syringic, cinnamic, and P-coumaric acids) was characterized against the five strains of interest. The antimicrobial properties of MPAs could not be measured directly using the multi-well plate assay. Low solubility due to increased hydrophobicity with introduction of the methyl chain resulted in sediment in the wells that blocked the travel of light through the sample, affecting measurements of bacteria density via absorbance. Therefore, antimicrobial properties of MPAs were quantified in terms log reduction of colony forming units (CFUs) relative to the control. The CFU results of MPAs in comparison with their PA controls are shown in Figure 8.

Modified BA (Figure 8A) had comparable activity to benzoic acid for all bacteria strains, except for drug-resistant *Staph. aureus*. Beyond potential complications with solubility, it is not clear why modified BA is less active than benzoic acid against the drug-resistant *Staph. aureus*; however, when comparing with the CA data in Figure 8C, the same trends can be observed. Namely, modified CA had comparable activity against all strains with the exception of drug-resistant *Staph. aureus*. These results indicate that the carboxylic acid end of BA and CA, both of which lack functional groups on the ring structure, may play a more significant role in antimicrobial activity against selected bacterial strains. Thus, modification of the carboxylic acid group for incorporation into polymer networks can negatively impact antimicrobial properties for specific applications and infection types. Previous work by Narasimhan et al. characterized a number of CA esters and amides against *E. coli* and *Staph. aureus* in addition to *B. subtilis*, *C. albicans*, and *A. niger* and found that all derivatives had comparable antimicrobial and antifungal activity to the CA control [40]. These results correlate with ours and provide further indication that modification of the carboxylic acid has strain-specific effects on antimicrobial activity.

Modified syringic acid had similar antimicrobial activity to syringic acid for all strains except *E. coli*, Figure 8B. It is not clear why this decrease was observed, but it could be partially related to poor solubility of the modified syringic acid in LB. As *E. coli* was more sensitive to DMSO than *Staph. epi.* and *Staph. aureus*, less DMSO could be used to aid in solubilizing the modified PAs in studies with *E. coli*. Reduced solubility may be related to decreased interactions between modified syringic acid and *E. coli*, which could have reduced antimicrobial efficacy. Modification of P-coumaric acid increased antimicrobial activity against *E. coli* and did not affect activity against the other tested strains, Figure 8D. This result correlates with previous work by Khatkar et al., wherein it was observed that substitution of the carboxylic acid of P-coumaric acid with bulky groups generally increased efficacy against *E. coli* [41]. It could be attributed to the increased hydrophobicity of modified P-coumaric acid that potentially increases its interactions with the hydrophobic lipid membrane on gram-negative *E. coli*. In general, this study demonstrates that PAs can be modified on the carboxylic acid end and retain their broad antimicrobial efficacy; however, tests should be conducted with specific strains of interest to confirm that desired antimicrobial properties are achieved after incorporation of PAs into synthetic biomaterials.

## 4. Conclusions

These studies provide the first systematic characterization of PAs in terms of structure and antioxidant and antimicrobial properties with an emphasis on antimicrobial efficacy against common wound and hospital-acquired pathogens. In general, hydrogen peroxide scavenging capabilities of PAs increase with increasing radical scavenging ring pendant groups with no trends observed between side chains. All tested PAs have high cytocompatibility, and the addition of multiple radical scavenging groups on the ring structure provides antioxidant efficacy to protect cells from cytotoxic levels of hydrogen peroxide. In terms of antimicrobial properties, all PAs showed efficacy against all tested strains. While there were some inconsistencies, a general trend was observed of decreased antimicrobial properties with increased ring hydroxyl and/or methoxy groups, which could be utilized in down-selection of functional PAs in future studies. The data collected on modified PAs indicates that the carboxylic acid groups on PAs can be reacted with biomaterial monomers without loss in antioxidant or antimicrobial functionality. This work provides a foundation for rational design of PA-based therapeutics on their own or as a part of biomaterial scaffolds with desired functionality. Future studies will utilize the same chemical modification of MPAs within a biomaterial network to provide biomaterials for wound healing with desired antioxidant and antimicrobial properties. These therapeutics could be used in a range of applications where antioxidant and/or antimicrobial properties are required for improved clinical outcomes.

## Figures and Tables

**Table 1:** Phenolic acid antimicrobial and antioxidant properties.

Phenolic Acid	Antimicrobial Properties		Antioxidant Properties
	Gram Positive	Gram Negative	
Cinnamic acid	*Staphylococcus epidermidis* *(Staph. epi.)* [12] *Staphylococcus aureus* *(Staph. aureus);* Multidrug-resistant *Staph. aureus* [11]	*Escherichia coli (E. coli)* [12] Multidrug-resistant *E. coli* [11]	N/A
P-coumaric acid	*Staph. aureus* [22] Methicillin-resistant *Staph. aureus* [24]	*E. coli* [25] Not effective against *E. coli* [22]	Relatively ineffective [16]
Ferulic acid	*Staph. aureus* [22]	*E. coli* [10] Not effective against *E. coli* [22]	0.9X trolox efficacy [16]
Sinapic acid	*Staph. aureus* [26]	*E. coli* [26]	6X trolox efficacy [16]
Caffeic acid	Methicillin-resistant *Staph. aureus* [24]	*E. coli* [10]	Scavenged 59% of H_2_O_2_ [15] 4X trolox efficacy [16]
Benzoic acid	Not effective against *Staph. aureus* [11]	Multidrug-resistant *E. coli* [11]	N/A
4-hydroxy-benzoic acid	*Staph. aureus* [27]	*E. coli* [10]	Relatively ineffective [16]
Vanillic acid	*Staph. aureus* [22] Methicillin-resistant *Staph. aureus* [24]	*E. coli* [10] Not effective against *E. coli* [22]	Relatively ineffective [16]
Syringic acid		Some efficacy against *E. coli* [25]	1.3X trolox efficacy [16]
Protocatechuic acid	Methicillin-resistant *Staph. aureus* [24,28]	*E. coli* [10,28]	Scavenged 55% of H_2_O_2_ [15] Inhibits macrophage production of O_2_ and H_2_O_2_ [29]
